# Genome Sequence of the Acute Urethral Catheter Isolate *Pseudomonas aeruginosa* MH38

**DOI:** 10.1128/genomeA.00161-14

**Published:** 2014-03-13

**Authors:** Daniel Wibberg, Petra Tielen, Jochen Blom, Nathalie Rosin, Max Schobert, Reinhilde Tüpker, Sarah Schatschneider, Dominik Spilker, Andreas Albersmeier, Alexander Goesmann, Frank-Jörg Vorhölter, Alfred Pühler, Dieter Jahn

**Affiliations:** a Institute of Microbiology, Technische Universität Braunschweig, Braunschweig, Germany; b Center for Biotechnology (CeBiTec), Universität Bielefeld, Bielefeld, Germany

## Abstract

*Pseudomonas aeruginosa* is a major nosocomial bacterial pathogen causing complicated catheter-associated urinary tract infections (CAUTIs). Here, we present the 6.9-Mb draft genome sequence of *P. aeruginosa* MH38 isolated from an acute nosocomial CAUTI. It exhibits resistance to several antibiotics but revealed low-level production of virulence factors.

## GENOME ANNOUNCEMENT

*Pseudomonas aeruginosa* is one of the most common Gram-negative nosocomial pathogens. Besides causing infections of the lungs, ears, and eyes, it is known as one of the major agents causing complicated catheter-associated urinary tract infections (CAUTIs) (1, 2). The production of a specific set of virulence factors and the enormous metabolic adaptability of *P. aeruginosa* often result in serious urinary tract infections (3).

Here, we describe the draft genome of *P. aeruginosa* MH38, isolated from an acute nosocomial CAUTI. The strain exhibits a nonmotile capsulated phenotype, with a low level of production of virulence factors but resistances to various antibiotics (3, 4).

In order to obtain its draft genome sequence, we extracted genomic DNA of *P. aeruginosa* MH38 to construct a paired-end library for shotgun sequencing on the Genome Sequencer FLX (GS FLX) system using Titanium technology (Roche), as described recently (5). The standard protocols were applied according to the manufacturer’s instructions. Assembly with the GS *de novo* Assembler software (Newbler) covered 216,518,852 bases from 958,125 aligned individual reads, with 345,167 paired-end reads among them. The assembly resulted in 141 contigs, which were organized in 9 scaffolds. The scaffolds consist of 6,889,973 bp, with an average coverage of 31.1× by shotgun reads. The genome has a G+C content of 65.83%.

Automated genome annotation was carried out using the GenDB software (6). This resulted in the prediction of 6,089 protein-coding sequences (CDSs). Five copies of the 5S, 16S, and 23S rRNA genes were identified, and 65 tRNAs were predicted. The genome sequence was compared with the core genome of *P. aeruginosa* using the software EDGAR (7). Thereby, 258 unique CDSs were identified in the MH38 genome. Among these, 59 phage- and 5 transposon-associated genes were found. Moreover, 3 genes involved in antibiotic resistance were predicted, plus 3 genes mediating metal resistance, 11 genes for regulators, 16 genes involved in metabolism, and 26 genes for virulence-related processes. Interestingly, a *trb* gene cluster encoding a type IV secretion system (P38_1269 to P38_1280, and P38_1979 to P38_1991), which is involved in the conjugal transfer of chromosomal and plasmid DNA in several Gram-negative bacteria (8, 9), was found. Moreover, genes were predicted for the autoinducer 2-degrading protein LsrG (P38_0246) and for synthetases of antimicrobial polypeptides, like bacitracin or gramicidin (P38_3486). Furthermore, additional genes may play a role in iron acquisition, such as a Fe^3+^-pyochelin receptor (P38_3479 to P38_3484) and the siderophore-interacting protein Sip (P38_6266). These proteins might contribute to meeting one of the major challenges in the urinary tract, the iron-limited environment (10).

The obtained genome sequence provides a solid basis for functional genomics analyses for a deeper understanding of *P. aeruginosa*-based CAUTIs.

### Nucleotide sequence accession numbers.

The sequence data related to this whole-genome shotgun project have been deposited in DDBJ/EMBL/GenBank under the accession no. CBTQ000000000. The version described in this paper is the first version, CBTQ000000000.1.
